# Predictors of Health-Protective and Helping Behaviors during the Covid-19 Pandemic: The Role of Social Support and Resilience

**DOI:** 10.1177/00332941221123777

**Published:** 2022-08-28

**Authors:** Andrea Fontes, Cícero Roberto Pereira, Sofia Menezes, Antonio Soares, Pedro Almeida, Geórgia Carvalho, Patrícia Arriaga

**Affiliations:** 56061 ISCTE-University Institute of Lisbon, BRU-IUL, Portugal; and 126808 Universidade Europeia, Lisbon, Portugal; Instituto de Ciências Sociais da Universidade de Lisboa, Lisbon, Portugal; and Universidade Federal da Paraiba, Brazil; Military Academy Research Center (CINAMIL); and 56061 ISCTE-University Institute of Lisbon, Portugal; 56061 ISCTE-University Institute of Lisbon, CIS_Iscte, Portugal

**Keywords:** Social support, helping behaviors, resilience, protective behaviors

## Abstract

The COVID -19 pandemic dramatically affected people’s lives. In this study, we explored the role of social and personal factors underlying individuals’ adaptive responses during the critical onset period of the outbreak. In particular, we tested two models on the mediating role of health-protective behaviors in the relationship between social support, resilience, and helping behavior. A sample of 1085 participants from Portugal and Brazil took part in an online survey during the first wave of the pandemic. First, through an Exploratory Factor Analysis of the health-protective behaviors to prevent contagion by the coronavirus, we identified two distinct dimensions, one aggregating active protective behaviors and the other as avoidant behaviors. Secondly, we found that resilience and active protective behavior sequentially mediated the relationship between social support and willingness to help. In addition, a multigroup analysis showed that this mediational process was similar in both countries. Given the wide range of social and individual factors that may predict prosocial behaviors, we highlight the role of social support on the intention to help through resilience and active protective behaviors.

## Introduction

In a message to the World Health Assembly in Geneva, Switzerland, the United Nations Secretary-General, António Guterres, stated that the Covid-19 pandemic is the greatest challenge of this era, classifying it as a tragedy (ONU News, 2020). Public health measures that interfere with personal freedoms and conflicting messages from authorities are stressors that may contribute to increased depressive and anxiety disorders (Pfefferbaum & North, 2020). The consequences have been felt in several domains. However, the social factor was one of the main impacted aspects: quarantine, social distancing, and self-isolation were identified as the most effective health-protective behaviors to reduce viral transmission. Nevertheless, social engagement, such as receiving and giving social support or engaging in prosocial behavior, also seems to mitigate the psychological harm that follows stressful circumstances (Flores et al., 2014) and may contribute to the well-being during the current pandemic (Miles et al., 2021). Thus, a better understanding of the contribution of social and individual factors to health-protective and prosocial helping behaviors is critical and was the main focus of our study.

### The Link Between Health-Protective and Prosocial Helping Behaviors

Health-protective behaviors were associated with prosociality in recent studies. Based on Caprara et al. (2012, p. 1289) definition, we will consider prosociality as “voluntary actions undertaken to benefit others, such as sharing, donating, caring, comforting, and helping”. For example, Franzen and Wöhner (2021) evidenced that support for preventive measures is one of the most important promoters of cooperation to prevent the spread of COVID-19. However, many countries implemented other public health and social measures to control the pandemic. Some required active health-protective behaviors, such as wearing masks or washing hands frequently.

In contrast, others were more related to social distancing by asking individuals to avoid face-to-face contact or stay at home. Adopting these measures may imply different behavioral-motivations related to protecting oneself from contracting the illness, preventing the spread of the disease, or protecting others (Banker & Park, 2020). In addition to these types of protecting behaviors, such as physical distancing, hygiene behaviors, and wearing a mask, Zickfeld et al. (2020) included prosocial behaviors when assessing health-protective behaviors in response to the COVID-19 outbreak. They justify the integration of prosocial behaviors in this assessment because they have considered all those protective behaviors as prosocial in the long run. Thus, in their view, adopting these behaviors is intertwined, assuming that health-protective behaviors such as physical distancing and hygiene predict prosociality. Other authors, however, took a different approach, differentiating the adoption of these measures more clearly by showing a distinct pattern of motivations. For example, Li et al. (2020) have demonstrated that health-protective and prosocial behaviors, although positively related, were weakly correlated.

On the other hand, while investigating prosociality among collectivist and individualistic countries, Zirenko et al. (2021) found that health-protective behaviors such as wearing a mask were chosen by those countries mainly for self-care reasons. Similar findings were reached by Banker and Park (2020) when analyzing the adoption of health-protective behaviors resulting from media messages. They conclude that the adoption of health-protective measures was more frequent when a self-focused frame (“protect yourself”) was used in media messages when compared with prosocial frame messages such as “protect your community”. Also worth mentioning are the studies showing that the perceived public threat about the virus was more strongly associated with prevention intentions than the perceived personal threat (Jordan et al., 2020; Lake et al., 2021).

Other researchers focused on the psychological factors behind the individual responses to preventive measures (Hartmann & Müller, 2022; Zajenkowski et al., 2020), namely personality traits. These studies evidence the positive relation between consciousness and neuroticism with risk avoidance, agreeableness with prosociality (Wilkowski et al., 2006), and more compliance with governmental restrictions (Zajenkowski et al., 2020). Hartmann and Müller (2022) also found that prevention regulatory focus was essential in adhering to and regulating preventive behavior besides agreeableness. Like Vaughn et al. (2020), these authors applied the regulatory focus theory to how people respond to COVID-19, distinguishing promotion from prevention regulatory focus.

However, the authors analyzed these variables as dispositional factors, not differentiating the public health preventive practices in active protective measures and social-avoidance responses. This distinction may be relevant if we consider the contemporary revision of the reinforcement sensitivity theory (RST; Gray & McNaughton, 2003), which explains how motivational systems become active in uncertainty or goal conflict situations. This theory distinguishes two motivational systems. The behavioral inhibition system (BIS) usually involves moving away from the threat with avoidant or isolation responses. The other is the behavioral approach system (BAS), which corresponds to defensive fight responses towards goal achievement. Thus, people might adhere differently to public health and social measures depending on their tendency towards avoidance/isolation or approach/active responses.

A recent study by Bacon and Corr (2020), with data collected in the first wave of the pandemic, has shown that both approach- and avoidance-related personality traits were significant predictors of behaviors due to the concerns about the coronavirus disease. Some people isolated themselves to cope with their fears, whereas others attempted to relieve uncertainty through approach behaviors, actively acting when facing a threat to re-establish their everyday life. In addition, these traits can also relate to different preferences for different forms of social support and prosocial behaviors (Bacon & Corr, 2020).

### The Role of Social Support and Resilience on Health-Protective and Prosocial Behaviors

The positive effect of social support on adopting health-protective behaviors has been studied before and during the current pandemic. Prior studies have shown that social support may lead to engagement in health-protective behaviors and encouragement and may increase self-esteem and sense of self-worth (Brown & Bond, 2008; Von Ah et al., 2004). It has been found, for example, that support from family has a positive relationship with greater adoption of general health practices (García-Huidobro et al., 2012), and support from peers can predict precautionary behaviors (Hurdle, 2001). Social support was a positive resource during the pandemic, which may contribute to adopting responsible behaviors such as health-protective self-care behaviors and other recommended actions to mitigate the spread of COVID-19 (Corral-Verdugo et al., 2021). In addition, the perception of a positive and close interpersonal environment, driven by social support, impacted the sense of belonging, which may also promote altruistic behaviors (de Guzman et al., 2012; Guo, 2017).

In contrast, social exclusion negatively impacted prosocial behaviors (Twenge et al., 2007). More recently, the relation between social support and prosociality has been found to also depend on the individual integration in the network plus the appraisal of the experienced social support (Drageset, 2021) and other individual factors, such as resilience.

Resilience refers to the capability to adapt and deal positively in adverse situations (Fletcher & Sarkar, 2013; Oliveira & Machado, 2011) and has been found to predict mental health and subjective well-being (Liu et al., 2013; Yu & Zhang, 2007). According to the risk-resilience model (Masten, 2001), the focus on adverse outcomes is enhanced in the face of adversity. However, resilient individuals seem to turn negative into positive outcomes (Yildirim & Arslan, 2020). In the current context, resilience has been found to display a protective function in reducing the negative effect of fear of COVID-19 (Seçer et al., 2020) and increasing subjective well-being and psychological health (Yildirim & Arslan, 2020).

Similarly, Kimhi et al. (2020) found a negative correlation between resilience and a sense of danger, aligned with the relations between resilience and risk perception (McCleskey & Gruda, 2021; Yildirim & Arslan, 2020).

However, results also suggest that the positive association between experience of fear and perceived risk can be helpful, as it may lead to engagement in preventive behaviors of being infected by the coronavirus. These behaviors include adopting social distancing and self-care measures such as handwashing (Harper & Rhodes, 2022; Vacondio et al., 2021; Yildirim & Arslan, 2020). Overall, the above findings might create some ambiguity about the role of resilience in the adoption of preventive behaviors: on the one hand, resilience is negatively related to risk perception, sense of threat, danger, and fear, whereas, on the other hand, these variables are positively correlated with health-protective behaviors.

Social support and resilience are also related. Bronfenbrenner’s bioecological theory (Bronfenbrenner, 1992) explained the relationship between social support and resilience by stating that individuals’ well-being depends on how individuals act and react to others and on the quality of their relationships with either family members or neighbors (Boon et al., 2012). The benefits of social support have been extensively studied in health promotion, coping abilities, and quality of life in both healthy and sick people (Eriksson & Lindström, 2008; Giebel et al., 2021). Social support has been considered an essential protective factor to promote resilience in challenging health contexts (Ferreira et al., 2018) and following catastrophic events (Hou et al., 2020; Rodriguez-Llanes et al., 2013). It helps to redefine adverse life events to be less threatening (Sippel et al., 2015) and with positive effects (on resilience) observed even 1 year after a natural catastrophe (Liu et al., 2013).

Moreover, social support and resilience proved to mediate the relationship between cognitive emotion regulation and acute stress responses (Cai et al., 2017). More recently, some studies conducted during the pandemic highlighted the role of social support as a contributor to higher resilience levels (Hou et al., 2020; Killgore et al., 2020; Mei et al., 2021). Killgore et al. (2020) found that social support from family, friends, and loved ones was associated with greater resilience during the lockdown. Drageset (2021) also supported this, identifying perceived social support as a potential resilience factor.

Our study will investigate the role of the above psychosocial constructs as predictors of health-protective and helping behaviors during the COVID-19 pandemic. We will explore the underlying structure of the measure of the health-protective behavior and investigate whether it will, in turn, predict helping behaviors. As predictors of these behavioral outcomes, we will include social and individual variables such as social support and resilience, which will be examined as independent predictors and components in a serial mediation model.

In addition, we will consider the country of residence as a potential moderator due to the contextual differences between the two countries that were the focus of our study: Brazil and Portugal. The governmental positions about the pandemic may influence the population’s behaviors differently, including prosocial precautionary behavior (Li et al., 2020), which may also affect the different diagnosed cases and mortality rates due to COVID-19 in these two countries.

For this reason, we studied two countries that adopted very different strategies during the COVID-19 pandemic. In Brazil, the federal government did not declare a state of emergency at the beginning of the outbreak. In the face of the increasing numbers of infected individuals, only some of the state governments decreed extended and mandatory social distancing. However, that did not occur uniformly in this country (Ribeiro, 2020). In contrast, the first state of emergency in Portugal was declared (Decree n.^º^2 - A/2020, 2020), imposing restrictions, containment, and preventive measures (Nunes, 2020). In addition, according to the “FM Global Resilience Index” (FM Global, 2021), these two countries also differ in resilience, with Portugal being positioned in 27th place in the ranking (76.8) and Brazil in 61st place (52.1).

### Objectives

Given that social support, resilience, and prosocial behavior were significantly correlated to well-being measures during the pandemic (Zhao et al., 2019), this study intends to shed light on the factors and mechanisms that can predict and contribute to explaining health-protective and helping behavior. Thus, our study has the following aims: (1) explore the structure of the health-protective behaviors that emerged during the COVID-19 outbreak by considering that the adoption of active health-preventive practices such as those related to hygiene measures, may be distinct from the avoidant behaviors related to social distancing recommendations; (2) examine how social support and personal resilience predict health-protective behaviors, which in turn predict helping behaviors; and (3) examine the potential moderation of the country where participants were living at the time the study was conducted.

**Figure 1.**
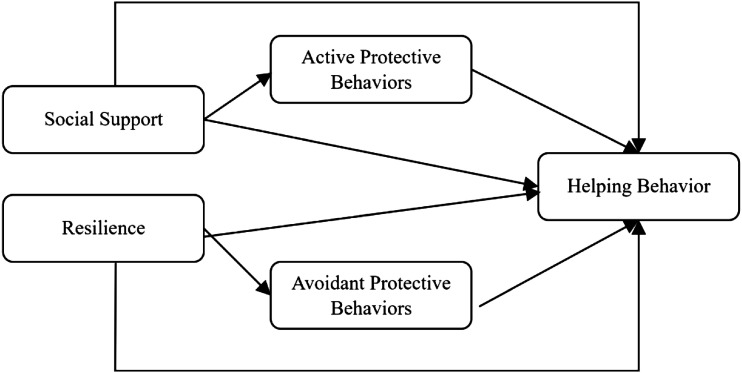
Model 1 representing social support and resilience as independent predictors of helping behavior through protective behaviors.

**Figure 2.**
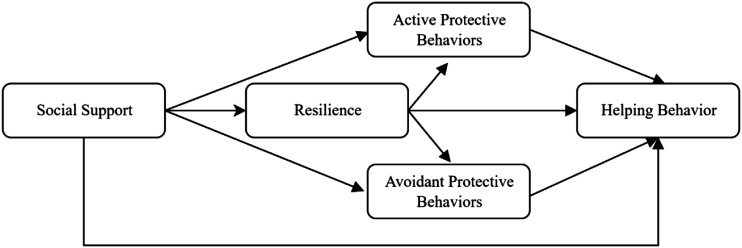
Model 2 representing resilience and protective behaviors sequentially mediating the relationship between social support and helping behavior.

We will examine the above relationships by the following analytical models (Figure 1 and Figure 2). As can be seen, the first model will test social support and resilience as independent predictors of helping behavior, with active protective behaviors and avoidant behaviors with distinct mediating processes. In the second model, resilience and individuals’ protective behaviors (differentiating active from avoidant behaviors) mediate the relationship between social support sequentially and helping behaviors sequentially.

## Method

### Participants

From an initial sample of 1729 participants, we removed from the analyses those who had missing values in our main variables (*n* = 631), failed the attention check (*n* = 272), and had residency out of Portugal or Brazil (*n* = 48). Our final sample is composed of 1072 participants, being 72.6% female (*n* = 778) and 27.1% male (*n* = 291). The remaining three participants refer to their gender as “other.” Participant’s ages ranged between 18 and 80 years (*M* = 38.08, *SD* = 12.74), with 52.6% living in Portugal (*n* = 564) and 47.4% in Brazil (*n* = 508).

### Measures and Procedure

After approval of the project by the local ethics committee, a survey was conducted online via the Qualtrics platform between 7 and 22 April 2020 and shared via social networks (Facebook, Instagram, WhatsApp, LinkedIn) and email. The survey started with informed consent and took 15 minutes to complete on average. We selected the most relevant scales and reduced formats when appropriate to reduce the participant’s burden in responding to a long survey.

For resilience, we used the Portuguese version of the subscale *personal competence* of the Resilience Scale (9 items) (Oliveira & Machado, 2011). The Resilience Scale was originally developed by Wagnild and Young (1993) and corresponded to the individual´s beliefs about their competences (e.g., “My belief in myself gets me through hard times”; “When I’m in a difficult situation, I can usually find my way out of it”). We asked the participants to respond to this scale while considering the pandemic context. Although the original response format has a 7-point scale, we used a 4-point scale (1 = *strongly disagree* to 4 = *strongly agree*) based on Johns’ (2005) suggestion that omitting the midpoint can improve the validity of the scale, especially when the topic may relate to social desirability. This resilience subscale has shown acceptable internal reliability (Cronbach’s α and McDonald’s ω = .86) in our study.

To evaluate Social Support, we used the eight-item of modified Medical Outcomes Study Social Support Survey (mMOS-SS) scale (Moser et al., 2012) with the items of the Portuguese version for this scale (Alonso Fachado et al., 2007). Participants were asked to indicate to what extent they would have the availability of someone to support them. Support was classified as instrumental/tangible (4 items, e.g., “to help you if you were confined to bed?”; “to take you to the doctor if you need it?”) and emotional (4 items; e.g., “to have a good time with?; “to turn to for suggestions about how to deal with a personal problem?”). In our study, internal reliability was very good for the overall social support score (Cronbach’s α = .92; McDonald’s ω = .93). The mean scores were computed, with higher scores indicating a higher perception of support from others during the pandemic.

As a way to evaluate health-protective behaviors, participants were asked to indicate how often they engaged in 13 behaviors since the COVID-19 outbreak. Most items were taken from the “precautionary behavior” measure developed by Li et al. (2020). However, this measure did not include all items (e.g., “change a face mask regularly”) because they were not recommended in the initial stages of the pandemic in the analyzed countries. Responses were given on a 5-point scale (1 = *never* to 5 = *always*). As we were adapting these health-protective behaviors to different countries, we ran an Exploratory Factor Analysis using the Principal Axis Factor extraction method and Oblimin rotation on the 13 precautionary health-protective behaviors to explore the underlying factor structure. We decided to conduct this exploratory analysis because we expected distinct patterns of health-preventive practices in response to government recommendations. Results suggested a two-factor solution with an eigenvalue higher than 1.00 (see Table 1). One of the factors was named *active protective behaviors* (APB). Factor APB aggregated eight items describing active behaviors and focusing on individual activities that participants could do for protection (e.g., “I monitor personal physical health”; “I am aware of my hygiene”; “I wash my hands”). The other factor was designated as *avoidant protective behaviors* (AVB). It aggregated five items describing avoidance of social contacts and related passive behavior, including reduced mobility of individuals. All five items were in line with the social distancing safety recommendations shared by local authorities to prevent the contagion by the coronavirus (e.g., “I avoid going to public establishments”; “I stay at home as long as possible”; “I avoid face-to-face contact with other people”). These two factors are slightly positively correlated (*r* = .22, *p* = .001), indicating their relatively independence, and showed sufficient internal consistency (Cronbach’s α = .68 and McDonald’s ω = .65 for active protecting behaviors; Cronbach’s α = .68 and McDonald’s ω = .69 for avoidant behaviors). The mean scores were computed for each dimension, with higher scores indicating higher active or higher avoidant protective behaviors to conform with the recommendation measures during the pandemic.

**Table 1.** Factor Loadings of Health-Protective Behaviors.

Items	Factor 1	Factor 2	Uniqueness
I monitor personal physical health	**.65**	−.07	.60
I am aware of my personal hygiene	**.54**	.05	.69
I monitor the physical health of people around me	**.53**	.03	.71
I wash my hands	**.46**	.08	.76
I try to maintain a balanced diet	**.44**	−.03	.81
I use a protective face mask	**.36**	−.02	.88
I use alcohol-based liquids	**.35**	.02	.88
I try to get enough sleep	**.31**	.08	.88
I avoid going to public establishments	−.04	**.68**	.55
I avoid moving to affected regions	−.02	**.55**	.70
I stay at home as long as possible	.01	**.60**	.64
I avoid face-to-face contact with other people	.14	**.49**	.71
I avoid traveling using public transports	.001	**.45**	.80
Eigenvalues	2.26	1.15	

Note. Principal axis factoring extraction method was used in combination with oblimin rotation.

To evaluate helping behavior intention, participants were asked about their willingness to participate in a set of five prosocial behaviors related to the COVID-19 pandemic during the following weeks. These five items were also taken from Li et al. (2020) and previously used in Oliveira et al. (2021) (e.g., “Dedicate time, donate money or supplies to chartered organizations or relevant institute”, “Elucidate others about the ways to deal with the current pandemic”, “Devote time to deliver goods and/or food to others”), and were evaluated on a 5-point scale. The scale ranged from 1 (*Never*) to 5 (*Very often*). The mean scores were also computed, given the acceptable internal reliability of this measure (Cronbach’s *α* = .70; McDonald’s *ω* = .69). Higher scores indicate higher intentions to help others during the pandemic.

Sociodemographic and health information included gender, education level, nationality, country of residence, and current perceived health condition. The items to measure health conditions were adapted from the Portuguese version of the European Social Survey (2018). Participants were asked to respond to the item “How is your health in general?”. Participants gave responses on a 5-point scale (1 = *Very good* to 5= *Very bad*) with the option to also state “*Don’t know*”.

## Results

### Preliminary Analysis

The correlation matrix of all main variables is presented in Table 2. Results indicated that social support was positively related to all variables. Resilience was associated positively with both social support and active protective behaviors. Notably, helping behaviors were correlated with most variables in the model and had a moderate association with active protective behaviors but a low relation with avoidant behaviors. Moreover, sociodemographic variables (gender, age) and perceived health were significantly related to helping behavior, active protective behaviors, and resilience. indicating that these variables should be controlled when testing the proposed serial mediation model.

**Table 2.** Correlations and Descriptive Statistics for Study Variables.

	(1)	(2)	(3)	(4)	(5)	(6)	(7)	(8)	(9)	(10)
Helping behavior (1)										
Active protective behaviors (2)	.37***									
Avoidant protective behaviors (3)	.09**	.22***								
Resilience (4)	.23***	.25***	−.003							
Social support (5)	.10***	.12***	.09**	.24***						
Gender^a^ (6)	−.13***	−.17***	−.14***	.07*	.04					
Age (7)	.10**	.25***	−.04	.19***	−.10***	−.01				
Education (8)	.17***	.13***	.03	.05	.05	−.07*	.15***			
Country^b^ (9)	−.16***	−.19***	.05	.10**	.04	.03	−.15***	−.19***		
Perceived health (10)	−.09**	−.17***	.01	−.32***	−.06	−.03	−.02	−11**	.09**	
*M*	2.77	3.88	4.62	3.13	3.91		38.09	5.76		1.94
*SD*	0.69	0.57	0.48	0.44	0.88		12.74	0.53		0.74

Note. **p < .01, ***p < .001.

^a^Gender was dummy coded (0 = women; 1 = men)

^b^Country of Residence was dummy coded (0 = Brazil; 1 = Portugal).

### Main Analysis

**Figure 3.**
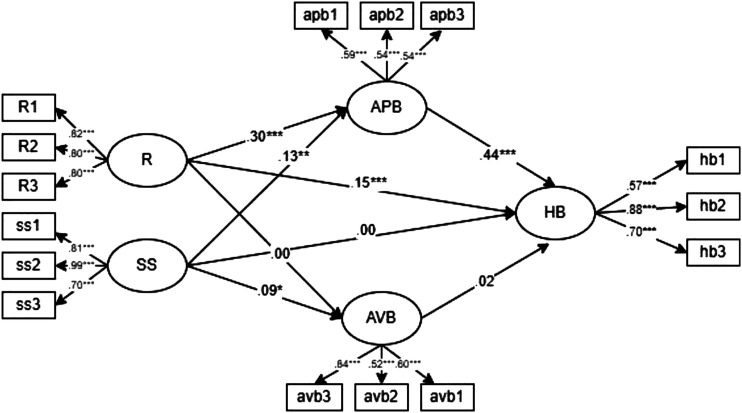
Standardized estimated parameters for the relation between social support and resilience, as independent variables, and helping behavior, mediated by protective behaviors. Note. *p < .05, **p < .01, ***p < .001; HB = helping behavior; SS = social support; R = resilience; APB = active protective behavior; AVB = avoidant behavior.

**Figure 4.**
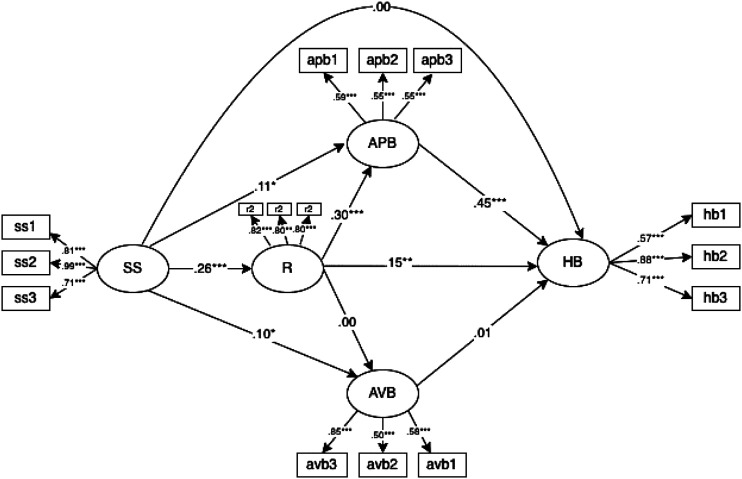
Standardized estimated parameters for the relation between social support and helping behavior, sequentially mediated by resilience and protective behaviors. Note. *p < .05, **p < .01, ***p < .001; HB = helping behavior; SS = social support; R = resilience; APB = active protective behavior; AVB = avoidant behavior.

We used the Mplus (version 8.6; Muthén & Muthén, 2019) to estimate two models specifying distinct hypotheses on the mediating role of protective behaviors in the relationship between social support, resilience, and helping behavior. The first model set social support and resilience as independent predictors of helping behavior, with active protective and avoidant behaviors proposed as mediators between social support and resilience and helping behavior. The second model specified a process in which resilience and individuals’ protective behaviors sequentially mediate the relationship between social support and helping behavior. Because we used several items to operationalize each variable, it was necessary to specify the latent factors being measured with three items parceling. This specification was the best measurement strategy because the items we used to measure each model variable are unidimensional in the current study (see section Method above). Parceling was considered the best option for estimating latent variables since it helps maintain the parsimony of the model and control for the measurement errors associated with the latent measurement factors (Little et al., 2002). We used the following conventional cutoff criteria to assess model fit (see Byrne, 2012 for a review): CFI and TLI higher than .95 indicate a good fit of the model to the data; RMSEA >.08 and indicates a misfit. In addition, we reported the χ2 likelihood ratio and associated degrees of freedom for descriptive information on model fit. Finally, we used the ∆χ2 to decide the best-fitting model, assuming a significant ∆χ2 indicative of a reliable difference between the models’ fitting to the data. Goodness-of-fit indices for the estimated model are presented in Table 3, and the standardized estimated parameters are presented in Figure 3 for Model 1 and Figure 4 for Model 2.

**Table 3.** Goodness-of-fit Indexes for the Tested Models.

Models	χ	df	χ2/df	CFI	TLI	RMSEA (90%CI)	AIC	Δχ2
Model 1 (Multiple Mediation)	356.05***	82	4.34	.94	.93	.056 (.050; .062)	29170	
Model 2 (Multiple Mediation)	315.50***	81	3.89	.96	.94	.052 (.046; .058)	29111	40.55***

Note. χ2 = chi-square test of model fit; df = degree of freedom; CFI = comparative fit index; TLI = tucker lewis index; RMSEA = root mean square error of approximation; Akaike Information Criterion; ΔCFI = difference between comparative fit indexes of two models; ***p < .001.

The results indicated that the two models fit the data well. However, Model 1 worked slightly below the cutoff criteria we specified for CFI and TLI, while Model 2 failed only on TLI. Moreover, Model 2 fitted significantly better than Model 1, as measured by Δχ2. Accordingly, the model specifying a serial mediation between social support and helping behavior presented slightly better empirical evidence to represent the relationships between study variables than Model 1, which predicted social support and resilience as two independent factors predicting helping behavior.

We used bootstrapping procedures with 5000 resamplings to estimate confidence intervals for total, direct and indirect effects (Table 4). Estimated parameters showed a reliable total effect between social support and helping behaviors, so the greater the social support, the more intention to help others the participants reported. The direct effect concerning this relationship was not reliable. Notably, the total indirect effect was reliably different from zero, indicating a sequential mediation. We decomposed this sequential indirect effect and found a reliable specific mediating effect between social support and helping behavior via resilience and active protective behaviors. Instead, the specific mediating effect through resilience and passive/avoidant protective behaviors was unreliable. These results suggest that active protective behaviors, but not avoidant and social isolation, played a mediating role in the model. Indeed, the greater the social support, the more participants self-reported resilience. And the greater the resilience, the greater the reports of active protective behaviors positively related to the intention to help others. (see Figure 4).

**Table 4.** Effects’ Decomposition of the Sequential Mediation Analyses.

		95% C.I.	
Effect	Estimate	Lower	Upper
Total effect	.13	.07	.19
Direct effect	.00	-.06	.06
Total indirect effect	.13	.09	.17
Specific indirect effects			
SS ⇒ APB ⇒ HB	.05	.02	.09
SS ⇒ AVB ⇒ HB	.00	-.01	.01
SS ⇒ R ⇒ HB	.04	.02	.06
Specific sequential indirect effects			
SS ⇒ R ⇒ APB ⇒HP	.03	.02	.05
SS ⇒ R ⇒ AVB ⇒HP	.00	.01	.01

Note. Estimates are standardized coefficients. HB = Helping Behavior; SS = Social Support; R = Resilience; APB = Active Protective Behavior; AVB = Avoidant Behavior.

We also found simple mediation effects. Specifically, the relationship between social support and intentions to display helping behavior was mediated by resilience (i.e., SS ---> R---> HB) and by active protective behaviors (i.e., SS ---> APB---> HB). These specific mediating effects indicated shorter routes through which social support can be related to helping behaviors, which did not go through the sequential process involving resilience and active protective behaviors.

### Complementary Analysis

We conducted a series of complementary analyses to investigate whether the country in which participants lived moderated a relationship between the proposed model variables. We then specified a serial model of moderated mediation using multigroup analysis. First, we estimated a baseline model in which the structural parameters were freely estimated between countries. The results showed an excellent fit to the data: χ^2^(df = 162; *N* = 1072) = 367.925, *p* <.001; χ^2^/gl = 2.27; CFI = .96; TLI = .94; RMSEA = .05 (90%CI: .04; .06). We then estimated a restricted model in which the structural parameters were fixed to equality and found an equally excellent fit to the data: χ^2^(df = 171; *N* = 1072) = 369.589, *p* <.001; χ^2^/gl = 2.16; CFI = .96; TLI = .95; RMSEA = .05 (90%CI: .04; .06). Importantly, the country did not moderate the serial mediation, we proposed to explain the relationship between the model variables, as the restricted model and the baseline model did not differ significantly (∆χ^2^ = 1.51, *p* = .99).

Due to the use of a correlational research design, the relationships between the protective and helping behaviors may have occurred in other ways. For example, these behaviors may be viewed as different expressions of social support, with resilience mediating these expressions. To account for this possibility, we estimated an alternative model in which protective behavior and helping behavior were specified as dependent variables at the same level in the model chain. Results showed a misfit to the data χ2(df = 83; *N* = 1072) = 403.556, *p* <.001; χ2/df = 4.86; CFI = .94; TLI = .93; RMSEA = .06 (90%CI: .05; .07). In addition, this alternative model fits worse than the proposed model 1 (Δχ^2^ = 47.51) and model 2 (Δχ^2^ = 88.06).

## Discussion

Considering the relevance of protective and prosocial behaviors in the current pandemic contexts, this study examined the role of social support and resilience in protective behaviors and the intention to help others. Results showed that social support was positively related to helping behavior, and this relation was serially mediated by resilience and active protective behaviors. Importantly, in the analysis of the two models tested, resilience was more effective as a mediator between social support and active protective behavior than as a sole predictor predicting that resilience, and in particular, the belief the individual has on his or her own competence, plays a stronger role in promoting helping behavior when predicted by social support. Furthermore, the multigroup analysis showed that this mediation process was similar in both countries.

### Different types of Protective Behaviors predicting Helping Behaviors through Resilience

Based on our results, we can conclude that not all health-protective behaviors to prevent the coronavirus spread and infection seem to predict helping behavior. Among the two types of protective behaviors that were analyzed, only the active protective behaviors related to hygiene and personal protection (such as wearing a mask or washing hands) worked as significant predictors of helping behavior, compared to avoidant protective behaviors characterized by social distancing and isolation to avoid the risk of contamination. Both types of precautionary practices were recommended to citizens during the early stage of the pandemic outbreak. Notwithstanding, the adoption of these measures might also be related to different motivations to behave when facing risk situations and, in particular, to individual differences in approach/avoidance motivational systems.

As mentioned by Bacon and Corr (2020, p. 846), “while some people will address their fears by isolating themselves, others (…) attempt to relieve uncertainty through approach behaviors”. Thus, the social distance recommendations might have been more adopted by individuals with stronger avoidant motivations when dealing with difficult situations. Additionally, since avoidance tends to be associated with the fear of contracting the disease (Bacon & Corr, 2020), we might assume that it can also limit the willingness to help others during the pandemic.

Analyzing the direct relation between social support and the two different protective behaviors we might see that social support predicts both active protective behaviors and avoidant behaviors. This prediction is in line with a recent manuscript with data collected among 69 countries during the pandemic, where it was found that avoiding physical contact and maintaining hygiene were related to prosociality (Pavlović et al., 2022). The authors identified social belonging as a relevant predictor of hygiene maintenance behaviors and physical distancing.

Suppose we add resilience as the predictor of both protective behaviors. In that case, we find that the belief in personal competence to manage difficult situations predicts the active protective behaviors positively, but not the avoidant behaviors. This suggests that a stronger belief in own’s competence predicts adherence to actively displaying precautionary hygiene and self-protection measures to face the pandemic, which, in turn, also increases their willingness to help others. In contrast, higher resilience was not related to avoidant and socially restrictive behaviors. Looking at the correlation between resilience and avoidant behaviors, even though non-significant, it presents an inverse direction. Somehow, this inverse direction might be explained by the protective function of resilience to reduce the negative effect of fear of COVID-19 (Seçer et al., 2020), adding information about the previously mentioned ambiguity about the role of resilience in the adoption of preventive behaviors. As an individual belief about competence, resilience might reduce the negative effect of fear by impelling to action, as evidenced in our study, through adopting protective behaviors, but not through avoidant behaviors.

In fact, according to what Bacon and Corr (2020) have shown, those who tend to isolate to avoid threats also tend to express greater fear of potential contamination. Thus, we might argue that this fear of contamination may lead to the limitation of social contacts to the minimum necessary, which we claim could limit prosocial motivations, at least the actions that imply personal contact.

### From Social Support to Helping Behaviors

Our results have shown that perceived social support is related to helping behaviors. Although the direct effect is small, these results are consistent with prior findings indicating that social support promotes altruistic behaviors (de Guzman et al., 2012; Guo, 2017). We might argue that during such a challenging context as the current pandemic, which somehow impacted all individuals, the same relation is found, and altruistic behaviors might be more activated. In addition, we found that the relation between social support and helping behaviors is serial mediated by resilience and active protective behaviors. We will further analyze each path of our model.

First, as expected, social support significantly predicted protective behaviors. Even though the government called for the need to practice “social distancing” (although the meaning seems to be “physical distancing”), social support was revealed as a contributor to the adherence to responsible and precautionary behaviors. This finding is consistent with recent findings showing the importance of social support as a resource by contributing to responsible protection behaviors during the pandemic (Corral-Verdugo et al., 2021; Pavlović et al., 2022).

Interestingly, the relation between social support and protective behaviors was statistically significant for active and passive protective behaviors, the two types of protective behaviors that emerged in our study. This result is somehow aligned with the recent literature mentioning that positive environmental factors stimulate responsible actions, such as precautionary behaviors, against COVID-19 (Corral-Verdugo et al., 2021).

Second, we also found that higher social support was related to greater resilience levels, consistent with prior studies (Hou et al., 2020; Liu et al., 2013; Rodriguez-Llanes et al., 2013; Sippel et al., 2015). Developing social relations and perceiving to receive social support seem to strengthen personal competencies to manage challenging situations, highlighting the importance of creating a reliable social support network to increase the sharing of feelings and problems (Killgore et al., 2020; Mei et al., 2021).

As recently mentioned by Hou et al. (2020, p. 9), “individuals with higher levels of social support believe that if they had to face a stressful event during the pandemic, they could get the help needed, seeing themselves as more prepared to deal with some adversity, meaning more their resilience reinforced.”. In our study, social support was strengthened as a predictor of resilience because the best fit for the tested models is found when resilience is used as a serial mediator. In this case, resilience is considered as opposed to being an independent predictor, and it demonstrates the specific role of social support in building resilience in the pandemic context.

Thirdly, the relationship between resilience and active protective behavior illustrates that resilience is not only an important trait that helps individuals overcome difficult moments and a variable that can promote engagement in active behavior to overcome the challenge, in this case, active protective behavior. In contrast, resilience negatively predicts passive/avoidant protective behavior, suggesting that those who perceive themselves as more resilient do not engage in avoidant behavior by isolating themselves. These findings, which suggest that resilience is related to adopting proactive but not avoidant behaviors, follow and complement the BIS/BAS classification applied to the pandemic context by Bacon and Corr (2020). Our findings are also consistent with the definition of resilience, characterized by personal traits such as persistence, optimism, and strength that facilitate individuals’ positive adaptation to negative contexts (Zhao et al., 2019). In this way, the findings contribute to resolving the previously identified dilemma regarding the role of resilience: either in contributing to the reduction of risk perception (McCleskey & Gruda, 2021; Yildirim & Arslan, 2020), making the event perceived as less catastrophic (Sippel et al., 2015) and consequently influencing the adoption of less protective behaviors or as contributing to better behavioral adaptation when dealing with challenging situations (Fletcher & Sarkar, 2013; Oliveira & Machado, 2011). Our findings support this second direction and expand the definition of resilience as the ability to actively find ways to better cope with adversity, in this case, by actively adopting protective behaviors. This way, resilience reinforces the protective role for well-being (Liu et al., 2013; Yu & Zhang, 2007) and promotes active protective behaviors. Consequently, resilience reinforces the intention to help others due to the predictive nature of the dynamic protective behaviors in prosociality (helping behavior) analyzed earlier.

Fourth, the predictive role of resilience in helping behaviors is enhanced, evidencing that resilience implies several approaches to face the pandemic obstacle. These approaches are not just related to the psychological way of facing constraints but also externalized in different behaviors to fight the threat, either through the engagement in active protection behaviors or the support of others who might need help.

Finally, we affirm that this support cycle can function as a virtuous circle, as those who receive more social support are also more likely to provide more support to others (de Guzman et al., 2012; Guo, 2017).

We consider this result significant in the COVID-19 pandemic, where social distancing was recommended, which could have translated into less access to social support and less willingness to help others. Notably, the country of residence was not a moderator of the model, suggesting that individual differences favoring these processes occurred despite the reality of the country and the restrictive measures imposed.

### Limitations and Future Studies

This study cannot be seen without limitations. The first limitation relates to the conclusions’ generalization with a sample collected in only two countries. Additionally, since we administered the survey to the participants electronically, we could not reach the population who do not have access to the Internet. However, we consider that the large sample size and the different realities that those countries were living in can somehow compensate for that effect. Also, being a cross-sectional study with data collected at the beginning of the pandemic, we captured just the first effects, with reduced literature and missing essential factors such as vaccination. Future studies should invest in collecting data in several moments and analyzing if some variables remain stable or suffer some changes. It would also be interesting for future studies to investigate the role of some personality traits in the model, the same way we tested social support and resilience. Future studies should analyze how personality traits predict helping behaviors, namely agreeableness and motivational predispositions, such as BIS and BAS. Since our study was focused on individual activities that participants could do for protection, we could not conceptualize the two dimensions as approach versus avoidance, which would be an interesting analysis. Similarly, we believe self-efficacy, as resilience, also able to be developed through interventions, could be analyzed as both an effect of social support and a predictor of helping behaviors.

## Conclusions and Practical Implications

The relevance of giving and receiving social support has been extensively explored in the literature in “normal” contexts but particularly in such challenging contexts as the current pandemic. One important conclusion is that the two types of protective behaviors recommended by local governments are distinct. Active protective behaviors like hygiene, wearing masks, and other actions directed to protect health predicted prosocial behaviors, whereas avoidant behaviors related to social distancing recommendations did not. This result suggests that although the avoidant behaviors were considered relevant to reducing the spread of COVID-19, they did not contribute to undertaking actions that would benefit others more directly. This fact could have happened because social distancing was a precautionary recommendation imposed with the confinement leading individuals to significantly change their usual behavior, considering others as a potential threat, having implications for prosociality. Based on our findings, we can conclude about the positive and significant contribution of social support, resilience, and active protective behaviors to prosociality, during the first quarantine. Resilience and active protective behaviors simultaneously mediate the relation between social support and helping behaviors, as well as their significant direct effects. Worth mentioning is the role of resilience in this model. Although it is considered a stable individual characteristic, it can also be developed through interventions (Albott et al., 2020). According to our model, the development of resilience is crucial not just to enhance the coping mechanisms of individuals under challenging contexts but also to favor the adoption of protective behaviors and positively predict the individual’s willingness to help others in times of need. Resilience also played an essential role in the explanatory mechanisms of the difference between active and avoidant protective behaviors, two claimed effective ways of mitigating the adverse effects of the pandemic, in particular in predicting active protective behaviors, but not the adherence to avoidant protective behaviors.

Based on our findings, we claim that authorities should reinforce the need to adopt protective behaviors in which the person displays a more active role. This is not only because these precautionary behaviors are relevant for health protection but also because adherence to these health practices seems more strongly related to helping others than adopting practices of social distancing and avoidance.

Lastly, we conclude that social support is beneficial to individuals, not just because it helps cope with the adversities through resilience enhancement but also for the possibility of leading to the adoption of protective and helping behaviors. These results emphasize the relevance of constructing more collaborative and socially supportive environments among restricted circles like family and friends and expanding into more comprehensive networks. We also believe that social support and prosociality are not just related but might create a virtuous circle of support, where both resilience and protective behaviors are involved.

## Supplemental Material

Supplemental Material - Predictors of Health-Protective and Prosocial Behaviors during the Covid-19 Pandemic: The Role of Social Support and Resilience Supplemental Material for Predictors of Health-Protective and Prosocial Behaviors during the Covid-19 Pandemic: The Role of Social Support and Resilience by Andrea Fontes, Cícero Pereira, Sofia Menezes, Antonio Soares, Pedro Almeida, Geórgia Carvalho, and Patrícia Arriaga in Psychological Reports

## References

[bibr1-00332941221123777] Albott C. S. Wozniak J. R. McGlinch B. P. Wall M. H. Gold B. S. Vinogradov S. (2020). Battle buddies: Rapid deployment of a psychological resilience intervention for health care workers during the COVID-19 pandemic. Anesthesia & Analgesia, 131(1), 43–54. 10.1213/ANE.0000000000004912 32345861 PMC7199769

[bibr2-00332941221123777] Alonso Fachado A. Montes Martinez A. Menendez Villalva C. Pereira M. G. (2007). Adaptação cultural e validação da versão Portuguesa Questionário Medical Outcomes Study Social Support Survey (MOS-SSS) [Cultural adaptation and validation of the medical outcomes study social support survey questionnaire (MOS-SSS)]. Acta Medica Portuguesa, 20(6), 525–534. https://www.actamedicaportuguesa.com/revista/index.php/amp/article/viewFile/894/568. 18331696

[bibr3-00332941221123777] Bacon A. M. Corr P. J. (2020). Coronavirus (COVID-19) in the United Kingdom: A personality-based perspective on concerns and intention to self-isolate. British Journal of Health Psychology, 25(4), 839–848. 10.1111/bjhp.12423 32348015 PMC7267391

[bibr4-00332941221123777] Banker S. Park J. (2020). Evaluating prosocial COVID-19 messaging frames: Evidence from a field study on Facebook. Judgment and Decision Making 15(6), 1037–1043. 10.2139/ssrn.3684901

[bibr5-00332941221123777] Boon H. J. Cottrell A. King D. Stevenson R. B. Millar J. (2012). Bronfenbrenner’s bioecological theory for modelling community resilience to natural disasters. Natural Hazards, 60(2), 381–408. 10.1007/s11069-011-0021-4

[bibr6-00332941221123777] Bronfenbrenner U. (1992). Ecological systems theory. In Vasta R. (Ed.), Six theories of child development: Revised formulations and current issues (pp. 187–249). Jessica Kingsley Publishers.

[bibr7-00332941221123777] Brown L. J. Bond M. J. (2008). An examination of the influences on health-protective behaviors among Australian men. International Journal of Men’s Health, 7(3), 274–287. 10.3149/jmh.0703.274

[bibr8-00332941221123777] Byrne B. (2012). Structural equation modeling with Mplus basic concepts, applications, and programming. Routledge.

[bibr9-00332941221123777] Cai W. Pan Y. Zhang S. Wei C. Dong W. Deng G. (2017). Relationship between cognitive emotion regulation, social support, resilience and acute stress responses in Chinese soldiers: Exploring multiple mediation model. Psychiatry Research, 256, 71–78. 10.1016/j.psychres.2017.06.018 28624675

[bibr10-00332941221123777] Caprara G. V. Alessandri G. Eisenberg N. (2012). Prosociality: The contribution of traits, values, and self-efficacy beliefs. Journal of Personality and Social Psychology, 102(6), 1289–1303. 10.1037/a0025626 21942280

[bibr11-00332941221123777] Corral-Verdugo V. Corral-Frías N. S. Frías-Armenta M. Lucas M. Y. Peña-Torres E. F. (2021). Positive environments and precautionary behaviors during the COVID-19 outbreak. Frontiers in Psychology, 12, 624155. 10.3389/fpsyg.2021.624155 33790838 PMC8006288

[bibr13-00332941221123777] de Guzman M. R. T. Jung E. Anh Do K. (2012). Perceived social support networks and prosocial outcomes among Latino/a youth in the United States. Revista Interamericana de Psicología, 46(3), 413–424. https://www.redalyc.org/pdf/284/28425871010.pdf

[bibr14-00332941221123777] Drageset J. (2021). Social support. In Haugan G. Eriksson M. (Eds.), Health promotion in health care – Vital theories and research (pp. 137–144). Springer International Publishing. 10.1007/978-3-030-63135-2_11 36315659

[bibr15-00332941221123777] Eriksson M. Lindström B. (2008). A salutogenic interpretation of the Ottawa Charter. Health Promotion International, 23(2), 190–199. 10.1093/heapro/dan014 18356285

[bibr16-00332941221123777] European Social Survey . (2018). ESS Round 9 Source questionnaire. University of London. https://www.europeansocialsurvey.org/docs/round4/fieldwork/source/ESS4_source_main_questionnaire.pdf

[bibr18-00332941221123777] Ferreira C. Guanilo M. E. E. Silva D. M. G. V. D. Gonçalves N. Boell J. E. W. Mayer B. L. D. (2018). Avaliação de esperança e resiliência em pessoas em tratamento hemodialítico. Revista de Enfermagem da UFSM, 8(4), 702–716. 10.5902/2179769230592

[bibr19-00332941221123777] Fletcher D. Sarkar M. (2013). Psychological resilience: A review and critique of definitions, concepts, and theory. European Psychologist, 18(1), 12–23. 10.1027/1016-9040/a000124

[bibr20-00332941221123777] Flores E. C. Carnero A. M. Bayer A. M. (2014). Social capital and chronic post-traumatic stress disorder among survivors of the 2007 earthquake in Pisco, Peru. Social Science & Medicine, 101, 9–17. 10.1016/j.socscimed.2013.11.012 24560219 PMC4083018

[bibr21-00332941221123777] FM Global Resilience Index . (2021). https://www.fmglobal.com/research-and-resources/tools-and-resources/resilienceindex (Accessed 31 July 2021).

[bibr22-00332941221123777] Franzen A. Wöhner F. (2021). Coronavirus risk perception and compliance with social distancing measures in a sample of young adults: Evidence from Switzerland. Plos One, 16(2), Article e0247447. 10.1371/journal.pone.0247447 33606826 PMC7894933

[bibr23-00332941221123777] García-Huidobro D. Puschel K. Soto G. (2012). Family functioning style and health: Opportunities for health prevention in primary care. British Journal of General Practice, 62(596), Article e198–e203. 10.3399/bjgp12X630098 PMC3289826 22429437

[bibr24-00332941221123777] Giebel C. Lord K. Cooper C. Shenton J. Cannon J. Pulford D. Shaw L. Gaughan A. Tetlow H. Butchard S. Limbert S. Callaghan S. Whittington R. Rogers C. Komuravelli A. Rajagopal M. Eley R. Watkins C. Downs M. Gabbay M. (2021). A UK survey of COVID-19 related social support closures and their effects on older people, people with dementia, and carers. International Journal of Geriatric Psychiatry, 36(3), 393–402. 10.1002/gps.5434 32946619 PMC7536967

[bibr25-00332941221123777] Gray J. A. McNaughton N. (2003). The neuropsychology of anxiety: An enquiry into the functions of the septo-hippocampal system. Oxford University Press. 10.1093/acprof:oso/9780198522713.001.0001

[bibr26-00332941221123777] Guo Y. (2017). The influence of social support on the prosocial behavior of college students: The mediating effect based on interpersonal trust. English Language Teaching, 10(12), 158–163. 10.5539/elt.v10n12p158

[bibr27-00332941221123777] Harper C. A. Rhodes D. (2022). Ideological responses to the breaking of COVID-19 social distancing recommendations. Group Processes & Intergroup Relations. Advance online publication. 10.1177/13684302221074546 PMC9922664 36816351

[bibr28-00332941221123777] Hartmann M. Müller P. (2022). Acceptance and adherence to COVID-19 preventive measures are shaped predominantly by conspiracy beliefs, mistrust in science and fear – A comparison of more than 20 psychological variables. Psychological Reports. Advance online publication. 10.1177/00332941211073656 PMC8883133 35212558

[bibr29-00332941221123777] Hou T. Zhang T. Cai W. Song X. Chen A. Deng G. Ni C. (2020). Social support and mental health among health care workers during coronavirus disease 2019 outbreak: A moderated mediation model. Plos One, 15(5), Article e0233831. 10.1371/journal.pone.0233831 32470007 PMC7259684

[bibr30-00332941221123777] Hurdle D. E. (2001). Social support: A critical factor in women’s health and health promotion. Health & Social Work, 26(2), 72–79. 10.1093/hsw/26.2.72 11379000

[bibr31-00332941221123777] Johns R. (2005). One size doesn’t fit all: Selecting response scales for attitude items. Journal of Elections, Public Opinion & Parties, 15(2), 237–264. 10.1080/13689880500178849

[bibr32-00332941221123777] Jordan J. Yoeli E. Rand D. G. (2020). Don’t get it or don’t spread it: Comparing self-interested versus prosocial motivations for COVID-19 prevention behaviors [Preprint]. PsyArXiv. 10.31234/osf.io/yuq7x PMC8511002 34642341

[bibr33-00332941221123777] Killgore W. D. S. Taylor E. C. Cloonan S. A. Dailey N. S. (2020). Psychological resilience during the COVID-19 lockdown. Psychiatry Research, 291, 113216. 10.1016/j.psychres.2020.113216 32544705 PMC7280133

[bibr34-00332941221123777] Kimhi S. Marciano H. Eshel Y. Adini B. (2020). Resilience and demographic characteristics predicting distress during the COVID-19 crisis. Social Science & Medicine, 265, 113389. 10.1016/j.socscimed.2020.113389 33039732 PMC7518838

[bibr35-00332941221123777] Lake J. Gerrans P. Sneddon J. Attwell K. Botterill L. Lee J. A. (2021). We’re all in this together, but for different reasons: Social values and social actions that affect COVID-19 preventative behaviors. Personality and Individual Differences, 178, 110868. 10.1016/j.paid.2021.110868 36540786 PMC9755896

[bibr36-00332941221123777] Li S. Wang Y. Xue J. Zhao N. Zhu T. (2020). The impact of COVID-19 epidemic declaration on psychological consequences: A study on active Weibo users. International Journal of Environmental Research and Public Health, 17(6), 2032. 10.3390/ijerph17062032 32204411 PMC7143846

[bibr37-00332941221123777] Little T. D. Cunningham W. A. Shahar G. Widamon K. F. (2002). To parcel or not to parcel: Exploring the question, weighing the merits. Structural Equation Modeling, 9(2), 151–173. 10.1207/S15328007SEM0902_1

[bibr38-00332941221123777] Liu Q. He F Jiang M. Zhou Y. (2013). Longitudinal study on adolescents’psychological resilience and its impact factors in 5.12 earthquake-hit areas. Journal of Hygiene Research, 42(6), 950–959. 24459907

[bibr39-00332941221123777] Masten A. S. (2001). Ordinary magic: Resilience processes in development. American Psychologist, 56(3), 227–238. 10.1037/0003-066X.56.3.227 11315249

[bibr40-00332941221123777] McCleskey J. Gruda D. (2021). Risk-taking, resilience, and state anxiety during the COVID-19 pandemic: A coming of (old) age story. Personality and Individual Differences, 170, 110485. 10.1016/j.paid.2020.110485

[bibr41-00332941221123777] Mei S. T. L. Ni A. O. Z. Sivaguru S. A/L Cong C. W. (2021). Social support, resilience, and happiness in response to COVID-19. Journal of Cognitive Sciences and Human Development, 7(1), 134–144. 10.33736/jcshd.2882.2021

[bibr42-00332941221123777] Miles A. Andiappan M. Upenieks L. Orfanidis C. (2021). Using prosocial behavior to safeguard mental health and foster emotional well-being during the COVID-19 pandemic: A registered report protocol for a randomized trial. Plos One, 16(1), e0245865. 10.1371/journal.pone.0245865 33503045 PMC7840018

[bibr43-00332941221123777] Moser A. Stuck A. E. Silliman R. A. Ganz P. A. Clough-Gorr K. M. (2012). The eight-item modified medical outcomes study social support survey: Psychometric evaluation showed excellent performance. Journal of Clinical Epidemiology, 65(10), 1107–1116. 10.1016/j.jclinepi.2012.04.007 22818947 PMC4119888

[bibr44-00332941221123777] Muthén L. K. Muthén B. (2019). Mplus user’s guide (8th Ed.). Muthén & Muthén.

[bibr45-00332941221123777] Nunes R. R. (2020). Ministra da Saúde diz que medidas de contenção serão necessárias até haver vacina [Minister of Health says containment measures will be necessary until there is a vaccine] Diário de Notícias. https://www.dn.pt/pais/mais-34-mortes-e-598-casos-de-covid-19-em-portugal-nas-ultimas-24-horas-12061197.html (Accessed 12 April 2020).

[bibr46-00332941221123777] Oliveira M. F. Machado T. S. (2011). Tradução e validação da Escala de Resiliência para estudantes do ensino superior [Translation and validation of the scale of Resilience for Students of Higher Education]. Análise Psicológica, 29(4), 579–591. 10.14417/ap.105

[bibr47-00332941221123777] Oliveira R. Baldé A. Madeira M. Ribeiro T. Arriaga P. (2021). The impact of writing about gratitude on the intention to engage in prosocial behaviors during the COVID-19 outbreak. Frontiers in Psychology, 12, 588691. 10.3389/fpsyg.2021.588691 33716858 PMC7943462

[bibr48-00332941221123777] ONU News . (2020). Covid 19 é o maior desafio desta era, diz Guterres à assembleia da OMS [Covid 19 is the biggest challenge of this era, said Guterres to the WHO assembly]. https://news.un.org/pt/story/2020/05/1713872 (Accessed 18 May 2020).

[bibr49-00332941221123777] Pavlović T. Azevedo F. Koustav D. Riaño-Moreno J. Maglić M. Gkinopoulos T. Donnelly-Kehoe P. Payán-Gómez C. Huang G. Kantorowicz J. Birtel M. Schönegger P. Capraro V. Santamaría-García H. Yucel M. Ibanez A. Rathje S. Wetter E. Bavel J. (2022). Predicting attitudinal and behavioral responses to COVID-19 pandemic using machine learning. PNAS Nexus, 1(3), pgac093. 10.1093/pnasnexus/pgac093 35990802 PMC9381137

[bibr50-00332941221123777] Pfefferbaum B. North C. S. (2020). Mental health and the Covid-19 pandemic. New England Journal of Medicine, 383(6), 510–512. 10.1056/NEJMp2008017 32283003

[bibr51-00332941221123777] Ribeiro J. R. (2020, April 29). Covid-19: No país onde o contágio é mais rápido, o Presidente responde: “E daí?” [Covid-19: In a country where the contagion is spreading faster, the President responds: “So what?”]. Público. https://www.publico.pt/2020/04/29/mundo/noticia/covid19-bolsonaro-lamenta-mortos-brasil-nao-faz-milagres-1914325

[bibr52-00332941221123777] Rodriguez-Llanes J. M. Vos F. Guha-Sapir D. (2013). Measuring psychological resilience to disasters: Are evidence-based indicators an achievable goal? Environmental Health, 12(1), 115. 10.1186/1476-069X-12-115 24359448 PMC3893382

[bibr53-00332941221123777] Seçer İ. Ulaş S. Karaman-Özlü Z. (2020). The effect of the fear of COVID-19 on healthcare professionals’ psychological adjustment skills: Mediating role of experiential avoidance and psychological resilience. Frontiers in Psychology, 11, 561536. 10.3389/fpsyg.2020.561536 33192830 PMC7609966

[bibr54-00332941221123777] Sippel L. M. Pietrzak R. H. Charney D. S. Mayes L. C. Southwick S. M. (2015). How does social support enhance resilience in the trauma-exposed individual? Ecology and Society, 20(4), 1–10. 10.5751/ES-07832-200410

[bibr55-00332941221123777] Twenge J. M. Baumeister R. F. DeWall C. N. Ciarocco N. J. Bartels J. M. (2007). Social exclusion decreases prosocial behavior. Journal of Personality and Social Psychology, 92(1), 56–66. 10.1037/0022-3514.92.1.56 17201542

[bibr56-00332941221123777] Vacondio M. Priolo G. Dickert S. Bonini N. (2021). Worry, perceived threat and media communication as predictors of self-protective behaviors during the COVID-19 outbreak in Europe. Frontiers in Psychology, 12, 577992. 10.3389/fpsyg.2021.577992 33664691 PMC7921485

[bibr57-00332941221123777] Vaughn L. A. Garvey C. A. Chalachan R. D. (2020). Need support and regulatory focus in responding to COVID-19. Frontiers in Psychology, 11, 589446. 10.3389/fpsyg.2020.589446 33329250 PMC7717948

[bibr58-00332941221123777] Von Ah D. Ebert S. Ngamvitroj A. Park N. Kang D.-H. (2004). Predictors of health behaviors in college students. Journal of Advanced Nursing, 48(5), 463–474. 10.1111/j.1365-2648.2004.03229.x 15533084

[bibr59-00332941221123777] Wagnild G. M. Young H. (1993). Development and psychometric evaluation of the resilience scale. Journal of Nursing Measurement, 1(2), 165–178. 7850498

[bibr60-00332941221123777] Wilkowski B. M. Robinson M. D. Meier B. P. (2006). Agreeableness and the prolonged spatial processing of antisocial and prosocial information. Journal of Research in Personality, 40(6), 1152–1168. 10.1016/j.jrp.2005.12.004

[bibr61-00332941221123777] Yildirim M. Arslan G. (2020). Exploring the associations between resilience, dispositional hope, preventive behaviors, subjective well-being, and psychological health among adults during early stage of COVID-19. Current Psychology, 41(8), 5712–5722. 10.1007/s12144-020-01177-2 33223782 PMC7666616

[bibr62-00332941221123777] Yu X. Zhang J. (2007). Factor analysis and psychometric evaluation of the Connor-Davidson Resilience Scale (CD RISC) with Chinese people. Social Behavior and Personality, 35(1), 19–30. 10.2224/sbp.2007.35.1.19

[bibr63-00332941221123777] Zajenkowski M. Jonason P.K. Leniarska M. Kozakiewicz Z. (2020). Who complies with the restrictions to reduce the spread of COVID-19? Personality and perceptions of the COVID-19 situation. Personality and Individual Differences, 166, 110199. 10.1016/j.paid.2020.110199 32565591 PMC7296320

[bibr64-00332941221123777] Zhao J. L. Cai D. Yang C. Y. Shields J. Xu Z. N. Wang C. Y. (2019). Trait emotional intelligence and young adolescents’ positive and negative affect: The mediating roles of personal resilience, social support, and prosocial behavior. Child & Youth Care Forum, 49(3), 431–448. 10.1007/s10566-019-09536-2

[bibr65-00332941221123777] Zickfeld J. H. Schubert T. W. Herting A. K. Grahe J. Faasse K. (2020). Correlates of health-protective behavior during the initial days of the COVID-19 outbreak in Norway. Frontiers in Psychology, 11, 564083. 10.3389/fpsyg.2020.564083 33123045 PMC7573186

[bibr66-00332941221123777] Zirenko M. Kornilova T. Qiuqi Z. Izmailova A. (2021). Personality regulation of decisions on physical distancing: Cross-cultural comparison (Russia, Azerbaijan, China). Personality and Individual Differences, 170, 110418. 10.1016/j.paid.2020.110418 33041413 PMC7538945

