# Supplementary material for: Predictors of Health-Protective and Helping Behaviors during the Covid-19 Pandemic: The Role of Social Support and Resilience
Source: Psychol Rep. 2022 Aug 28;127(6):2736–61. doi: 10.1177/00332941221123777 (PMC11528867; doi:10.1177/00332941221123777)
Supplement: Supplemental Material - Predictors of Health-Protective and Prosocial Behaviors during the Covid-19 Pandemic: The Role of Social Support and Resilience [file sj-pdf-1-prx-10.1177_00332941221123777.pdf]

Table S1.

Effect decomposition of sequential mediation analyses after accounting for control variables.

		**95% C.I.**	
**Effect**	**Estimate**	**Lower**	**Upper**
Total Effect	.13	.07	.19
Direct Effect	-.02	-.08	.04
Total Indirect Effect	.15	.11	.20
Specific Indirect Effects:			
SS ⇒ APB ⇒ HB	.07	.03	.10
SS ⇒ AVB ⇒ HB	.00	-.01	.01
SS ⇒ R ⇒ HB	.05	.03	.08
Specific Sequential Indirect Effects:			
SS ⇒ R ⇒ APB ⇒ HB	.03	.02	.05
SS ⇒ R ⇒ AVB ⇒ HB	.00	-.01	.01

Note. Estimates are completely standardized coefficients. HB = Prosocial Behaviour; SS = Social Support; R = Resilience; APB = Active Protective Behaviour; AV = Avoidance Behaviour.
Control variables= country of residence (dummy: 0 = Brazil; 1 = Portugal), perceived health status, and sociodemographic data (gender: 0 = women, 1 = men; education; and age)
